# Advances in De Novo Drug Design: From Conventional to Machine Learning Methods

**DOI:** 10.3390/ijms22041676

**Published:** 2021-02-07

**Authors:** Varnavas D. Mouchlis, Antreas Afantitis, Angela Serra, Michele Fratello, Anastasios G. Papadiamantis, Vassilis Aidinis, Iseult Lynch, Dario Greco, Georgia Melagraki

**Affiliations:** 1 Department of ChemoInformatics, NovaMechanics Ltd., Nicosia 1046, Cyprus; papadiamantis@novamechanics.com; 2 Faculty of Medicine and Health Technology, Tampere University, 33520 Tampere, Finland; angela.serra@tuni.fi (A.S.); michele.fratello@tuni.fi (M.F.); dario.greco@tuni.fi (D.G.); 3 BioMEdiTech Institute, Tampere University, 33520 Tampere, Finland; 4 School of Geography, Earth and Environmental Sciences, University of Birmingham, Birmingham B15 2TT, UK; I.lynch@bham.ac.uk; 5 Institute for Bioinnovation, Biomedical Sciences Research Center Alexander Fleming, Fleming 34, 16672 Athens, Greece; Aidinis@fleming.gr; 6 Institute of Biotechnology, University of Helsinki, 00014 Helsinki, Finland; 7 Finnish Center for Alternative Methods (FICAM), Tampere University, 33520 Tampere, Finland; 8 Division of Physical Sciences & Applications, Hellenic Military Academy, 16672 Vari, Greece

**Keywords:** de novo drug design, artificial intelligence, machine learning, deep reinforcement learning, artificial neural networks, recurrent neural networks, convolutional neural networks, generative adversarial networks, autoencoders

## Abstract

De novo drug design is a computational approach that generates novel molecular structures from atomic building blocks with no a priori relationships. Conventional methods include structure-based and ligand-based design, which depend on the properties of the active site of a biological target or its known active binders, respectively. Artificial intelligence, including ma-chine learning, is an emerging field that has positively impacted the drug discovery process. Deep reinforcement learning is a subdivision of machine learning that combines artificial neural networks with reinforcement-learning architectures. This method has successfully been em-ployed to develop novel de novo drug design approaches using a variety of artificial networks including recurrent neural networks, convolutional neural networks, generative adversarial networks, and autoencoders. This review article summarizes advances in de novo drug design, from conventional growth algorithms to advanced machine-learning methodologies and high-lights hot topics for further development.

## 1. Introduction

The development of a chemical entity and its testing, evaluation, and authorization to become a marketed drug is a laborious and expensive process that is prone to failure [1]. Indeed, it is estimated that just 5 in 5000 drug candidates make it through preclinical testing to human testing and just one of those tested in humans reaches the market [2]. The discovery of novel chemical entities with the desired biological activity is crucial to keep the discovery pipeline going [3]. Thus, the design of novel molecular structures for synthesis and in vitro testing is vital for the development of novel therapeutics for future patients. Advances in high-throughput screening of commercial or in-house compound libraries have significantly enhanced the discovery and development of small-molecule drug candidates [4]. Despite the progress that has been made in recent decades, it is well-known that only a small fraction of the chemical space has been sampled in the search for novel drug candidates. Therefore, medicinal and organic chemists face a great challenge in terms of selecting, designing, and synthesizing novel molecular structures suitable for entry into the drug discovery and development pipeline.

Computer-aided drug design methods (CADD) have become a powerful tool in the process of drug discovery and development [5]. These methods include structure-based design such as molecular docking and dynamics, and ligand-based design such as quantitative structure–activity relationships (QSAR) and pharmacophore modeling. In addition, the increasing number of X-ray, NMR, and electron microscopy structures of biological targets, along with state-of-the-art, fast, and inexpensive hardware, have led to the development of more accurate computational methods that accelerated the discovery of novel chemical entities. However, the complexity of signaling pathways that represent the underlying biology of human diseases, and the uncertainty related to new therapeutics, require the development of more rigorous methods to explore the vast chemical space and facilitate the identification of novel molecular structures to be synthesized [6].

De novo drug design (DNDD) refers to the design of novel chemical entities that fit a set of constraints using computational growth algorithms [7]. The word “de novo” means “from the beginning”, indicating that, with this method, one can generate novel molecular entities without a starting template [8]. The advantages of de novo drug design include the exploration of a broader chemical space, design of compounds that constitute novel intellectual property, the potential for novel and improved therapies, and the development of drug candidates in a cost- and time-efficient manner. The major challenge faced in de novo drug design is the synthetic accessibility of the generated molecular structures [9]. In this paper, advances in de novo drug design are discussed, spanning from conventional growth to machine learning approaches. Briefly, conventional de novo drug design methodologies, including structure-based and ligand-based design using evolutionary algorithms, are presented. Design constraints can include, but are not limited to, any desired property or chemical characteristic, for example: predefined solubility range, toxicity below a threshold, and specific chemical groups included in the structure. Finally, machine-learning approaches such as deep reinforcement learning and its application in the development of novel de novo drug design methods are summarized. Future directions for this important field, including integration with toxicogenomics and opportunities in vaccine development, are presented as the next frontiers for machine-learning-enabled de novo drug design.

## 2. De Novo Drug Design Methodology

**Figure 1:**
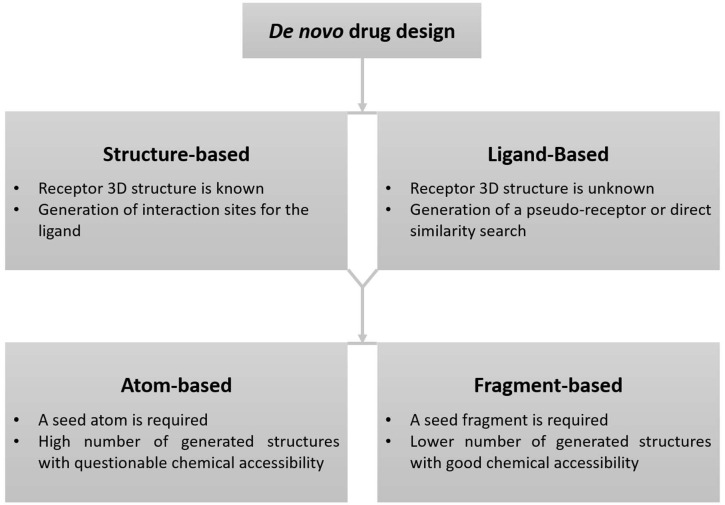
Schematic representation of the de novo drug-design methodology.

De novo drug design is a methodology that creates novel chemical entities based only on the information regarding a biological target (receptor) or its known active binders (ligands found to possess good binding or inhibitory activity against the receptor) [10,11,12,13,14]. The major components of de novo drug design include a description of the receptor active site or ligand pharmacophore modeling, construction of the molecules (sampling), and evaluation of the generated molecules. Two major de novo drug-design approaches are available including structure-based and ligand-based design (Figure 1). The three-dimensional structures of a receptor are generally available through X-ray crystallography, NMR, or electron microscopy [15,16]. When the structure of the receptor is unknown, homology modeling can be employed to acquire a suitable structure for de novo drug design [17]. However, the quality of a homology model depends on the quality of the template structure and sequence similarity. The Ligand-based approach is generally used when no structural data for the biological target are available, but instead one or more active binders are known [3].

### 2.1. Structure-Based De Novo Drug Design

Receptor-based de novo drug design begins with defining the active site of the receptor. Since the molecular shape, physical, and chemical properties of the active site are important for tight and specific binding of a ligand, the active site is analyzed to determine the shape constraints and the non-covalent interactions for a ligand [9]. Receptor–ligand non-covalent interactions consist of hydrogen-bonds, electrostatic, and hydrophobic interactions and are used to generate interaction sites for a ligand. These sites play a significant role in reducing the high number of generated structures, thus increasing selectivity. There are several methods used to define interaction sites for the active site of the receptor. An example is HSITE, a rule-based method which considers only hydrogen-bond donors and acceptors generating a map of hydrogen-bonding regions [18]. LUDI and PRO_LIGAND are other ruled-based methods that also consider hydrophobic interaction sites [19,20,21]. HIPPO is a ruled-based method that considers the interaction sites of covalent bonds and metal ion bonds [22]. Other methods include grid-based approaches, in which a grid of points is generated in the active site of the receptor, and interaction energies for hydrogen-bonding or hydrophobic interactions are calculated using probe atoms or fragments at each grid point [23,24,25]. Multiple-copy simultaneous search (MCSS) is a method that randomly docks functional groups in the active site to determine energetically favorable positions and orientations [26,27]. The functional groups are then minimized using a force-field, and groups are discarded if the interaction energy between them and the active site is not favorable based on a threshold value. The evaluation of the candidate structures is important in de novo drug design and it is generally performed by calculating the free binding energy of the candidate molecule with the binding site of the receptor using scoring functions. The main scoring functions used to evaluate the generated structures include force fields, empirical scoring functions, and knowledge-based scoring functions [28,29,30,31,32].

### 2.2. Ligand-Based De Novo Drug Design

When the three-dimensional structure of a biological target is absent, known active binders offer an alternative strategy for de novo drug design [3]. Such data are available in the literature from screening efforts or structure–activity relationship studies [33]. Active binders can also be found in databases such as ChEMBL, which contains bioactive molecules with drug-like properties [34]. This method is often employed to design novel candidate structures for biological targets for which obtaining a crystal structure is challenging [35]. From one or more known active binders, a ligand pharmacophore model is established and used to design novel structures. In particular, the ligand pharmacophore model can be utilized either to create a pseudo-receptor or to directly perform similarity design [21]. It is worth mentioning that the quality of the pharmacophore model plays a significant role in ligand-based de novo drug design and it depends on the structural diversity of the known binders. The possibility of different binding modes requires the assumption of a common binding mode to build the pharmacophore model. A quantitative structure–activity relationship model can be used in parallel to evaluate the quality of the pharmacophore model [36]. Examples of ligand-based de novo drug design tools include TOPAS [37], SYNOPSIS [38], and DOGS [39].

### 2.3. Sampling Methods in De Novo Drug Design

Sampling of the candidate structures can be achieved by two methods, namely atom-based and fragment-based approaches [8,9]. In atom-based sampling, an initial atom is randomly placed in the active site and used as a seed to construct the rest of the molecule. In every stage, a variety of atoms and hybridization states of each atom are explored. As a result, the chemical space covered by this method is vast and the generated structures need to be narrowed down. This is typically achieved by filtering the structures based on chemical accessibility. Atom-based sampling has the advantage of a higher exploration of the chemical space, and thus a greater number and variety of structures are generated. However, the high number of generated structures makes it difficult to identify suitable compounds for synthesis and experimental testing. LEGEND is an example of an atom-based de novo drug-design algorithm [24]. Fragment-based sampling is the preferred method in de novo drug design because the structures are generated as fragment assemblies, which narrows the chemical search space, maintains good diversity, and generates candidate compounds with chemical accessibility and optimal adsorption, distribution, metabolism, excretion and toxicity (ADMET) properties [8]. This method requires a database that contains fragments and linkers, which are obtained either virtually or experimentally [3]. A fragment is docked in the active site and is utilized as a seed to build the rest of the molecule [40,41,42]. Examples of algorithms that employ fragment-based design as a sampling method include LUDI [43], PRO_LIGAND [20], SPROUT [44], and CONCERTS [29]. It is worth mentioning that drug properties such as ADMET can be implemented in de novo drug design using secondary target constraints [9]. For example, structures with drug-like properties such as oral bioavailability can be obtained by filtering the proposed structures using Lipinski’s rule of five or other in silico predictive models [45,46,47].

## 3. Evolutionary Algorithms in De Novo Drug Design

**Figure 2:**
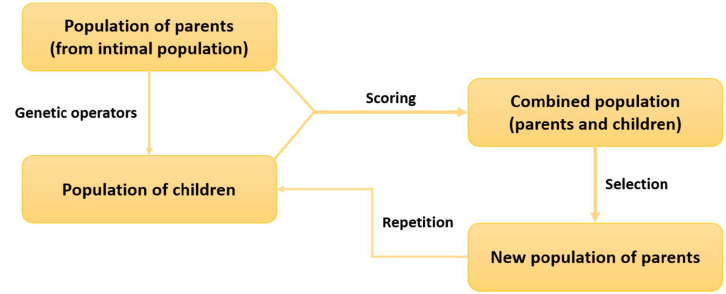
Schematic representation of the evolutionary algorithmic cycle in de novo drug design.

Evolutionary algorithms have been extensively used in de novo drug design [8]. These algorithms are subdivided into genetic algorithms, genetic programming, evolutionary programming, and evolutionary strategies, which are based on population optimization using mechanisms inspired by biological evolution, such as reproduction, mutation, recombination (crossover), and selection [48,49]. In the case of drug design, a population of structures or conformations is created, and each member of the population is encoded by a randomly generated chromosome. The cycle begins with the generation of a “parent” population from a randomly (stochastically) created initial population (Figure 2). Each parent undergoes a random transformation using genetic operators to generate a population of new structures, called “children”. The two principal operators used are mutation and crossover. Mutation generates new populations by introducing new information, while crossover uses this information to create new individual populations of the candidate structures. A fitness function is then employed to evaluate the binding score of each “child” structure. Based on the score, a new generation of parents is selected from the combined population of the initial “parents” and “children”. This new population of “parents” is used in the next cycle. This cycle is repeated until the termination criterion is fulfilled [50,51,52]. The main evolutionary techniques used in de novo drug design include genetic algorithms, evolutionary strategies, and evolutionary graphs [8]. Examples of de novo drug-design applications using genetic algorithms include LigBuilder [25], LEA [53], ADAPT [54], PEP [55], SYNOPSIS [38], LEA3D [56], GANDI [40] and ML GAN [57]. De novo drug-design tools utilizing evolutionary strategies are TOPAS [37], Flux(1) [58], and FLUX [59]. Finally, examples of de novo drug-design applications employing evolutionary graphs are MEGA [60] and EvoMD [61].

## 4. Artificial Intelligence in De Novo Drug Design

**Figure 3:**
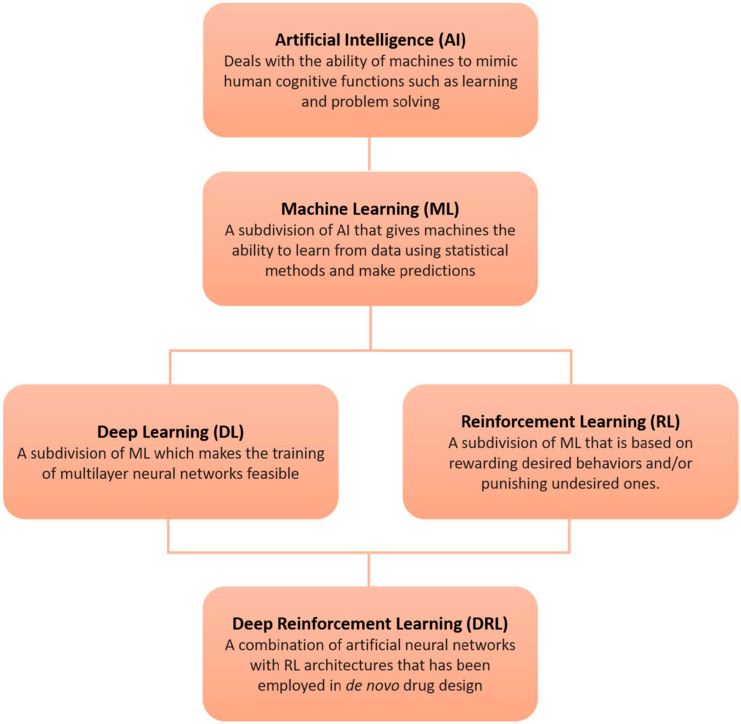
Artificial intelligence methods such as machine, deep, and reinforcement learning have been successfully employed in de novo drug design.

Artificial intelligence (AI) is a scientific field that exploits the ability of machines to mimic human cognitive functions such as learning and problem solving (Figure 3) [62,63,64,65]. Machine learning (ML) is a subdivision of AI that enables machines to learn from data using statistical methods and to make predictions [66,67]. ML methods have been employed to predict outcomes related to drug discovery [68]. Deep learning (DL) is a subdivision of ML which makes the computation of multilayer neural networks feasible [69]. The increased volumes of data available, combined with continuous increasing computer power, gave rise to DL methods such as recurrent neural networks (RNN), convolutional neural networks (CNN), generative adversarial networks (GAN), and autoencoders (AE). Reinforcement learning (RL) is another subdivision of machine learning, based on rewarding desired behaviors and/or punishing undesired ones [70]. Deep reinforcement learning (DRL) is a combination of artificial neural networks with reinforcement learning architectures, and has recently been employed in de novo drug design [71,72]. Such methods are expected to revolutionize the field of drug discovery since they are remarkably successful in other fields including recognition of speech [73], formal languages [74], video representations [75], music [76], and more.

### Deep Reinforcement Learning (DRL) in De Novo Drug Design

**Figure 4:**
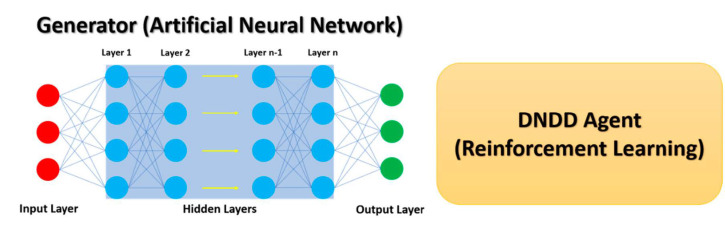
Deep reinforcement learning consists of a generator which is usually an artificial neural network and a de novo drug-design agent that uses reinforcement learning to make decisions for the generation of novel molecular structures.

Among the range of AI subdivisions, DL has been very popular in mimicking human abilities of image recognition and natural language processing [77]. In addition, DL has been employed for the development of analysis approaches in data-driven fields such as biomedicine and healthcare [78,79]. In drug discovery, DL was initially employed for the development of QSAR to predict properties such as affinity, toxicity, etc. [80,81]. Advances in drug discovery DL methods led to the development of fully connected neural networks using molecular descriptors calculated directly from molecular structures [82]. De novo drug design using DRL, which combines artificial neural networks with reinforcement learning, is a breakthrough in the field of drug discovery [72,83]. DRL approaches in de novo drug design typically consist of a generative model (generator) and a de novo drug-design agent that uses reinforcement learning (Figure 4). For the generative model, a multilayer artificial neural network is used. Depending on the type of artificial network, the input layer might consist of SMILES or graphs of molecules [84]. SMILES represents a molecule as a sequence of characters corresponding to atoms and special characters denoting connectivity [85]. The neural network is then trained using tokens of pre-existing data such as known bioactive molecules for a specific biological target. Construction of output structures is a result of iterative learning and decision-making steps [83]. At each step, the model determines the optimal token from the vocabulary based on the generated sequence of previous steps. The de novo drug design agent is part of the reinforcement framework, and it could be conceptualized as a virtual robot that interacts with molecules and modifies them to improve their properties. The actions of the agent are controlled by the artificial neural network, also called the generator.

## 5. Examples of DRL in De Novo Drug Design

### 5.1. Recurrent Neural Networks (RNN)

A recurrent neural network (RNN) is an artificial neural network architecture that employs cyclic connections between neurons [86,87]. These connections enable an RNN to have an inner representation of the current state, which enables it to remember information from previous steps in a sequence. Because of that, an RNN is suitable for the analysis of sequential data such as text or molecules represented as a sequence of characters like SMILES. RNN works sequentially by processing one step at a time in a series of actions. RNN can learn from SMILES strings’ patterns and the molecules produced from the de novo molecule procedure are chemistry-driven.

RNN combined with reinforcement learning was successfully employed in the de novo drug design of novel molecular entities [88,89,90]. The first step of this method includes a fine-tuned RNN that is pre-trained using existing bioactive molecules from a database such as ChEMBL. Training of an RNN is generally performed through maximum likelihood estimations of the next token in a target sequence of given tokens from the previous steps [72]. Once the RNN has been trained on target sequences such as SMILES, it is then used to generate new sequences that follow the conditional probability distributions learned from the training set [72]. In the second step, a de novo drug-design agent is generated based on a policy that maps a state to the probability of each action taken. Based on a set of actions taken from states and the received rewards, the agent policy is improved to increase the expected return. Two approaches have been used in reinforcement learning to obtain a policy: policy-based RL in which a representation of the decision policy is explicitly built and kept in memory during learning, or a value-based RL where only a value function is stored while the policy is implicit. A task that has a clear endpoint is referred to as an episodic task, which in the case of de novo drug design, is the generation of a SMILES string for a novel molecular entity.

Several examples of DRL in de novo drug design that employed RNN were reported in the literature, including a model that was trained to generate sulfur-free molecules using augmented episodic likelihood [72]. Reinforcement Learning for Structural Evolution (ReLeaSE) is an application of DRL to the problem of designing chemical libraries with the desired physicochemical and biological properties [91]. This approach uses a special type of stack-augmented RNN that was successful in inferring algorithmic patterns. This implementation considers SMILES strings as sentences composed of characters used in SMILES notation. The objective of stack-RNN is to learn the hidden rules of forming sequences of letters that correspond to legitimate SMILES strings. SMILES strings are used for both generative and predictive phases of the method and these phases are integrated into a single workflow. A fragment-based DRL approach, based on an actor-citric model, for the automatic generation of molecules with improved properties, was developed using RNN and RL [92]. This model learns how to modify molecules to improve their properties by generating novel structures that are similar to existing bioactive compounds of a given target. Thus, this approach does not attempt to search the entire chemical space to find optimal candidate molecules; instead, it optimizes an existing lead compound by adding fragments.

A multi-objective evolutionary de novo drug-design approach was developed using RNN to generate novel molecules [93]. The best molecules were selected to retrain the network using transfer learning (TL). In TL, a model is trained on a source task and then retrained on a new related task called the target task [94]. TL has been proven to be efficient in improving the accuracy of models based on narrowly defined tasks. A deep learning methodology using a long short-term memory (LSTM) RNN was successfully employed in de novo drug design [95]. The first part of this study involved training an LSTM-based RNN model to generate libraries of valid SMILES strings with high accuracy. TL was then used to fine-tune the model by generating molecules that are structurally similar to drugs with known bioactivities against a particular biological target. This method was found to be successful in the early stages of drug discovery where there is a low amount of data available. The second part of this study involved the application of the generative model to fragment-based drug discovery by growing a library of leads starting from a known active fragment [95].

An interesting study demonstrated that molecular information, such as molecular descriptors, can be incorporated into a conditional RNN generative process [96]. The generation process of this approach was conditioned with properties calculated either directly from molecular structures or QSAR, such that the encoder part was no longer needed. The conditional seed successfully steered the focus of the RNN towards a particular subset of the chemical domain, such as bioactive compounds of a biological target. A novel way of assessing the focus of a probabilistic sequence generator was also achieved using negative log-likelihood plots. An RNN trained on large sets of molecules was employed to develop a data-driven de novo drug-design approach [89]. This study demonstrated that an RNN trained on SMILES strings of molecules can both learn the grammar required to generate valid SMILES and generate molecules with similar properties to the compounds used for the training of the RNN [97]. A recent study assessed bidirectional molecule generation with RNN, comparing three bidirectional strategies (novelty, chemical biological relevance, and scaffold diversity) to the unidirectional forward RNN approach for the computer-generated molecules with SMILES string generation [98].

### 5.2. Convolutional Neural Networks (CNN)

A convolutional neural network (CNN) is a type of artificial network consisting of altering, convolution, and pooling layers, which enables them to extract features automatically [99,100,101]. CNNs were extensively employed in image processing with great success by running a small window over the input feature vector at both training and test phases as a feature detector [77]. This process allows a CNN to learn various features of the input regardless of their absolute position within the input feature vector [99]. DeepScaffold is a comprehensive solution for scaffold-based de novo drug design that utilizes CNN and 2D graphs of molecular structures [102]. This method can generate molecules based on a wide spectrum of scaffold definitions including Bemis–Murcko scaffolds, cyclic skeletons, and scaffolds with specifications of side-chain properties. An advantage of this method is its ability to generalize the chemical rules of adding atoms and bonds to a given scaffold. The compounds generated by DeepScaffold were evaluated by molecular docking to their associated biological targets, and the results suggested that this approach could be effectively applied in drug discovery. DeepGraphMolGen is a multi-objective computational strategy for generating molecules with desirable properties using a graph CNN and reinforcement learning [103]. This strategy consists of property prediction and molecular generation in which molecules were represented as 2D graphs, since they are a more natural molecular representation than SMILES strings. Finally, a new framework for de novo drug design was proposed based on a graph generation model. The graph generator was designed to be suitable for the task of molecule generation using a simple decoding scheme and a graph convolutional architecture that is less computationally expensive [104].

### 5.3. Generative Adversarial Networks (GAN)

A generative adversarial network (GAN) is a special type of neural network model where two networks are trained simultaneously, with one focused on image generation and the other centered on discrimination [105,106,107]. The generator typically captures the distribution of true examples for new data example generation. The discriminator is usually a binary classifier, discriminating the generated examples from the true examples as accurately as possible. GANs have been found to be successful in image generation tasks, including text-to-image synthesis, super-resolution, and image-to-image translation [105]. An original deep neural network (DNN) architecture called a reinforced adversarial neural computer (RANC) was utilized for de novo drug design of novel small-molecule organic structures based on a GAN and reinforcement learning [108]. RANC uses a differentiable neural computer, a category of neural network with increased generation capabilities due to the addition of an explicit memory bank mitigating common problems found in adversarial settings, as the generator. RANC was able to generate structures that match the distributions of key chemical descriptors and the lengths of SMILES strings in the training dataset [108]. Adversarial threshold neural computer (ATNC) is another de novo drug-design approach based on a GAN architecture and reinforcement learning. This approach uses a differentiable neural computer as the generator and has a new specific block, called an adversarial threshold, which acts as a filter between the agent (generator) and the environment (discriminator and objective reward factions) [109]. To generate more diverse molecules, a new objective reward function, named internal diversity, clustering (IDC) was employed [109]. LatentGAN is a novel deep-learning architecture which combines an autoencoder and a GAN for de novo drug design [110]. The utility of this method was examined using two scenarios: the first to generate random drug-like compounds and the second to generate target-biased compounds, with promising results in both cases. This method generates molecules that differ from those obtained using RNN-based generative models, indicating that these two approaches are complementary [110].

### 5.4. Autoencoders (AE)

#### 5.4.1. Variational Autoencoder (VAE)

A variational autoencoder (VAE) is a stochastic variational inference and learning algorithm that is extensively used to represent high-dimensional complex data via a low-dimensional latent space learned in an unsupervised manner using encoders and decoders [111]. De novo drug-design approaches using VAE include the development of a method to convert discrete representations of molecules to a multidimensional continuous representation [112]. In this study, a DNN was trained on hundreds of thousands of existing chemical structures to construct three coupled functions: an encoder, a decoder, and a predictor. The encoder converts the discrete representation of a molecule into a real-valued continuous vector, and the decoder converts these continuous vectors back into discrete molecular representations. The predictor estimates chemical properties from the latent continuous vector representation of the molecules. This model allowed efficient exploration of the chemical space through the development of optimized chemical structures. A shape-based generative approach for de novo drug design was developed using CNN to generate novel molecules from a seed compound, its three-dimensional shape, and its pharmacophoric features [113]. A VAE is used to perturb the 3D representation of a compound, followed by a system of convolutional and recurrent neural networks that generate a sequence of SMILES tokens. The generative design of novel scaffolds and functional groups performed by this method could cover unexplored regions of the chemical space that still possess lead-like properties. A conditional VAE was employed to develop a new molecular design strategy that directly produces molecules with the desired target properties [114]. This method controls multiple target properties by imposing them onto a condition vector. The authors demonstrated that it was possible to generate drug-like molecules with specific values for the five target properties (molecular weight (MW), octanol–water partition coefficient (LogP), number of hydrogen bond donors and acceptors (HBD, HBA) and topological polar surface area (TPSA)) within an error range of 10%. In addition, the authors were able to selectively control LogP without changing the other molecular properties and to increase a specific property beyond the range of the training set [114].

#### 5.4.2. Sequence-to-Sequence Autoencoder (seq2seq AE)

A sequence-to-sequence autoencoder (seq2seq AE) is an artificial network architecture that maps an input sequence to a fixed-sized vector in the latent space using a gated recurrent unit (GRU) [115] or an LSTM network [116], and then maps the vector to a target sequence with another GRU or LSTM network [117]. Thus, the latent vector is an intermediate representation containing the “meaning” of the input sequence. In the case of de novo drug design, the input and output sequences are both SMILES strings [118]. A generative network complex was successful in generating new drug-like molecules based on multi-property optimization via a gradient descent in the latent space of an autoencoder [118]. In this approach, both multiple chemical properties and similarity scores were optimized to generate drug-like molecules with the desired properties. The predictions of this method were validated using independent two-dimensional predictors based on molecular fingerprints. Finally, the method was utilized to generate a large number of new BACE1 inhibitors, as well as thousands of novel alternative drug candidates for eight existing drugs currently on the market, including Ceritinib, Ribociclib, Acalabrutinib, Idelalisib, Dabrafenib, Macimorelin, Enzalutamide, and Panobinostat [118]. A seq2seq AE was also used to develop a de novo drug-design approach using SMILES strings [119]. Using this method, the extent to which translation between different chemical representations influences the latent space similarity to the SMILES strings or circular fingerprints was explored. It was found that training a seq2seq hetero-encoder based on an RNN with LSTM cells to predict different enumerated SMILES strings from the same canonical SMILES string gives the largest similarity between latent space distance and molecular similarity measured as circular fingerprints similarity [119].

#### 5.4.3. Adversarial Autoencoder (AAE)

An adversarial autoencoder (AAE) is a probabilistic autoencoder that uses a GAN to perform variational inference by matching the aggregated posterior of the hidden code vector of an autoencoder with an arbitrary prior distribution [120]. An AAE that contains both a generator and a discriminator was trained on a set of molecules with anti-tumor growth activity [121]. The generated model was utilized to create molecules with the desired properties in the form of fingerprints. A close examination of the newly generated molecules showed that the newly created molecular fingerprints matched the structure of highly effective anticancer drugs. An improvement in this architecture led to the development of druGan, an AAE that incorporated additional molecular properties such as solubility and allowed the generation of molecules with different chemical structures [122]. druGan showed better performance in terms of feature extraction, ability to generate molecules, and reconstruction error compared to its predecessor AAE.

## 6. Particle Swarm Optimization for De Novo Drug Design

Particle swarm optimization (PSO) is a stochastic optimization technique inspired by swarm intelligence, which aims to find an optimal point in a search space defined by an objective function. The particle swarm consists of individual agents (particles) that optimize the given problem in parallel by making use of knowledge gained during its search. Additionally, particles constantly exchange information about optimization successes, and thus influence their direction of movement in the search space. During the search, promising solutions are identified in the region that attracts most of the particles. PSO has been successfully applied in the field of drug design for optimization of the molecular properties of compounds with the desired biological properties. For example, Hartenfeller et al. developed COLIBREE, an algorithm for fragment-based molecular de novo drug design based on PSO optimization [123]. In their approach, PSO guides the process of combinatorial de novo drug design. The constructed molecules follow a fixed build-up scheme with three main elements: (1) a user-defined molecular scaffold, which is used as the starting point for the molecular design; (2) building blocks which are molecular fragments derived by pseudo-retrosynthesis from known bioactive molecules; (3) linkers that represent substructures connecting two building blocks and link the building blocks to the scaffold. The whole design process, including the selection of linkers, building blocks, and structure assembly, is controlled by PSO, where each particle of the swarm identifies new candidate compounds. These compounds are evaluated by a ligand similarity-based fitness function to measure how far the new drug is from a reference set of drugs in a topological atom-pair description space. The particles in the swarm gain knowledge during the process, which is incorporated into the choice of new building blocks and linkers until a final optimal solution is reached.

Winter et al. developed a computational methodology that integrates PSO with in silico prediction of molecular properties such as biological activity and pharmacokinetics [124]. They used a DNN to learn a compressed latent space representation of compounds from 75 million chemicals. They defined a fitness function for the identification of the best candidate drug as a combination of structure–activity-relationship knowledge (for example, fixed ranges for molecular weight, and number of hydrogen-bonds.), a set of targets that should be hit by the compound, and pharmacokinetics-related properties. The PSO is used to search the compounds’ latent space and identify the optimal candidate drug. The main advantage of this approach is that the optimization can be performed using multiple objective functions simultaneously. However, the authors pointed out that this methodology has to be used in combination with constraints to the part of the chemical space that can be modeled within a reasonable applicability domain.

## 7. Evaluation Criteria

The design of new compounds is only the first step in the development of new drugs and is followed by an iterative loop of synthesis, analysis, and molecular optimization. However, not every generated compound can undergo this resource-intensive process. Therefore, it is necessary to focus the efforts on a few promising de novo generated compounds. For a compound to be relevant, it has to reach a balance between several contrasting aspects, which include the right amount of novelty: it should not be too similar to known drugs but also not too different so as to be completely unpredictable; it has to be stable and synthesizable; it should be feasible to produce; and it should score highly in the prediction of its desired properties (for example, target affinity and drug-likeness).

### 7.1. Diversity and Novelty

Generative models usually produce a population of chemical compounds on the order of hundreds or thousands of generated samples. Of course, not every compound will be unique; inevitably, some generated compounds will share characteristics to a lesser or greater degree, and some others will be more similar to the training data, or the reference database, depending on the specific method. It is especially important to test the capability of producing a wide variety of new structures when working with deep generative models, as failure may happen where the generated samples lack variety (such as mode collapse in GANs [125]) or the generated samples resemble the mean of the training distribution too much (blurry samples produced by AE-based models). To explore the similarity of a group of generated compounds against the reference set, several similarity scores have been proposed depending on the representation format of the molecules. The edit (or levenshtein) distance can be used to evaluate how different two SMILES strings are. If the molecules are represented as substructure fingerprints, such as extended-connectivity circular fingerprints (ECFP) or molecular access system (MACCS) keys, the Tanimoto and Dice distances can be used. Finally, several graph similarity measures (or graph kernels) have been proposed for evaluating the similarity of molecules represented by graphs, such as the random walk kernel or the convolutional kernel [126].

Due to the novelty of these methods, standardized approaches do not yet exist when it comes to specific workflows and validation guidelines for computer-assisted drug development, such as, for example, the Organization for Economic Cooperation and Development (OECD) rules for the validation of QSAR models [127]. Similarly, no guidelines currently exist on the acceptable ranges of evaluation metrics. This means that the selection of good evaluation thresholds for new generated drugs is subjective, experiential, and domain-specific and the decision is left to the human operator. As discussed in the next session, a current workaround would be the application of well-established ADMET and QSAR approaches, prior to synthesis and in vitro testing, for assessing the relevance of the computer-designed molecules, as also stated by Muratov et al. [128]. In any case, further work in this direction is required to identify specific criteria that computer-generated compounds must fulfill, even if these are still subject to the same in vivo and in vitro tests of a usual drug-discovery process.

### 7.2. Desired Properties

Virtually all the generative approaches rely on an evaluation mechanism that rewards some aspects of the generated compounds, such as target affinity or desired bioactivity. However, there are other “side” properties that, during the generating process, evolve in a substantially unconstrained manner. Moreover, these side properties may represent the difference between the success and failure of the development stages following the design and synthesis of a candidate drug molecule. Examples of undesired side properties include low drug-likeness scores [129] and undesired binding affinity with other complexes, which may reduce the overall efficacy or even cause adverse effects and cellular toxicity. Thus, in order to focus the synthesis efforts on the most promising generated compounds, a prioritization mechanism is often employed. The easiest approach is filtering and/or ranking of the generated molecules according to predicted drug-likeness, first introduced by Lipinski et al. [45], or ADMET properties [130], such as the solubility of the molecule, permeability of the brain–blood barrier, and affinity to transport proteins [126].

### 7.3. Synthetic Feasibility

Another concern is the actual capability of synthesizing the most promising de novo generated compounds for further evaluation and optimization [6,18,22,131,132]. Left unconstrained, generative models may propose overly complex or even impossible-to-produce compounds. The generative process can be biased by penalizing the complexity of the molecules, but at the expense of reduced efficacy [133]. Several evaluation functions have been proposed to estimate the complexity of a generated compound, like the synthetic accessibility (SA) score, which takes into account the presence of non-standard structural features, such as large rings, non-standard ring fusions, stereocomplexity, and molecule size [131], and the synthetic complexity (SC) score, which was trained using a reaction corpus based on 12 million reactions from the Reaxys database to impose a pairwise inequality constraint to ensure that reaction products are more synthetically complex than their corresponding reactants [134]. In addition to filtering the results of the learning process, the ability to generate realistic compounds can also be enforced directly at the level of the generation process. For example, the SPROUT algorithm assigns a different penalty to each fragment while assembling the compounds based on a database of fragments with known complexity [135]. The requirement of synthetic feasibility can be used as an inductive bias while training a deep generative model to only design synthetically feasible compounds. For example, MoleculeCHEF incorporates knowledge of basic reactants and a chemical reaction prediction model, and generates molecules through a series of simulated reactions [135]. Thus, each generated sample is expressed as a bag of base reactants and a “recipe” of chemically stable reactions, which are supposed to produce the target compound.

## 8. Bridging Toxicogenomics and Molecular Design

Toxicogenomics is the field of study that links the safety assessment of chemicals to the underlying biological mechanisms [136,137]. One important aspect tackled by toxicogenomics is the characterization of the mechanism-of-action (MOA) of a compound, represented as the set of all molecular alterations induced by the exposure of an organism (human) to it. Elucidation of the MOA allows understanding of the chain of biological events (such as immune system activation, changes in the metabolism, and effects to the cell cycle) triggered by a specific chemical (drug) exposure, which will lead to a phenotypic endpoint (for example, toxicity). Merging the cheminformatic and toxicogenomic methods, in combination with DL techniques, would facilitate and speed up the development of novel approaches where chemicals are designed de novo to exert specific molecular alterations and phenotypic effects.

Most of the approaches proposed to date are chemocentric, but new methodologies that bridge toxicogenomics and molecular design are starting to emerge. For example, Mendez-Lucio et al. developed a DL model based on a GAN whose training was conditioned by gene expression data [138]. In a conditional generative model, target properties for each compound are incorporated into the training and generative phases, in addition to the compound chemical representation. Thus, conditional models learn a latent representation space, which is a good representation of the compounds and of the conditional variables. This makes the models useful both for reconstruction and predictive tasks. By using this kind of approach, the trained model can be used to generate new compounds with a predicted transcriptomic alteration similar to the one required in the input. This approach, compared with more traditional similarity search-based approaches, has the main advantage of not being limited to the initial pool of compounds for which the gene expression signature is measured. Indeed, generative models can also help overcome the limitation of the chemical space by generating new compounds tailored to match the query gene expression signature. However, further work is required to assess the optimal biological models in which to generate the gene expression signatures, especially in the light of the variability of drug responses in cell lines, and the well-known limitations (relative advantages and disadvantages) of utilizing cell lines versus primary cells.

## 9. De Novo Drug Design for COVID-19

The coronavirus SARS-CoV-2 is responsible for the ongoing COVID-19 pandemic. The novel nature of this virus urgently requires the development of efficient drug repositioning and de novo drug-design approaches. The scientific community has been actively working in this field and some of the well-known AI-based methods for drug design have been applied to generate new compounds [139,140,141]. For example, Ton et al. developed a novel DL platform, called deep docking, that provides fast prediction of docking scores for structure-based virtual screening of billions of molecules simultaneously [142]. They displayed their application by applying the deep docking method to more than one billion compounds from the ZINC15 library and found 1000 potential ligands for the SARS-CoV-2 main protease (M^pro^) protein. These candidate inhibitors are chemically diverse and have superior docking scores compared to known protease inhibitors.

Chenthamarakshan et al. have developed a new method, called CogMol, for target-specific drug design for COVID-19 using deep generative models [143]. They first trained a VAE to learn the SMILES representations of the molecules. Then, they used a pre-trained protein sequence embedding from 24 million Uniprot protein sequences to train a protein-molecule binding affinity regressor that they used to guide the generation of new molecules. Finally, CogMol is empowered with an in silico screening protocol for the generated molecules, which accounts for factors such as the toxicity prediction of a clinical endpoint and the synthetic feasibility, and performs docking calculations to estimate the binding of the generated molecules to target proteins. They used the CogMol framework to generate candidate molecules to bind three relevant targets of the SARS-CoV-2 spike protein with high affinity. From the generated drugs that passed the in vitro screening filters less than 20 compounds, for each of the three protein targets considered, match an existing SMILES in PubChem. Among them are Plasmepsin-2 and Plasmepsin-4 inhibitor, ACE-2 inhibitors, and drugs approved for skin diseases and pneumonia. Since these drugs have already been approved for specific uses, it should be faster to have them approved for the treatment of COVID-19.

A different approach was applied by Tang et al. [144], who developed an advanced deep Q-learning network with fragmented-based drug design for generating potential lead compounds targeting the SARS-CoV-2 3C-like M^pro^. Their approach starts from a molecular fragment library built from a starting set of 284 molecules knowing to inhibit the SARS-CoV-2 3C-like M^pro^. Next, they applied an advanced deep Q-learning network, which combines meaningful molecular fragments, for generating new candidate compounds. They generated 4922 unique valid structures. Among these, 47 were selected by their reward function (for example, how the agent “ought” to behave) and further evaluated with docking and covalent docking studies [144].

Bai et al. developed a new tool for 3D drug design of protein targets, called MolAICal [145]. This tool combines deep generative models based on Wasserstein GAN (WGAN) and virtual screening to generate new compounds starting from a library of fragments from US FDA-approved drugs. They used MolAICal to generate new drugs targeting the membrane protein glucagon receptor (GCGR) and the non-membrane protein SARS-CoV-2 M^pro^. They used 21,064 fragments of FDA-approved drugs extracted from the e-Drug3D database and 1,060,000 drug-like ligands obtained from the ZINC database, and showed that MolAICal can generate various ligands with high 3D structural similarity to the crystal ligand of GCGR or SARS-CoV-2 M^pro^.

## 10. Building Community and Regulatory Acceptance of DL Methods for De Novo Drug Design

These COVID-19 examples demonstrate the power of DL methods for de novo drug design and are likely to further accelerate the drug discovery pipeline and the repurposing of existing drugs against alternative pathologies in the coming decade. However, since the development of DL-based de novo drug design approaches is still at an early stage, experimental validation of its effectiveness in drug discovery is crucial for the continuous improvement of these methods and to support their widespread uptake into medicinal chemistry practice and drug regulation. A recent report from the European Medicines Agency (EMA) and Heads of Medical Agencies (HMA) on regulatory challenges from big data suggested that “Algorithm code should be more transparent (feature selection, code, original data set) and available for targeted review by regulators. Outcomes of, and changes to, algorithm use (safety and efficacy) need to be subject to post-marketing surveillance mechanisms, just like it is done today to monitor drug safety after marketing authorization” [146]. A first key step is complete documentation of the DL models and the underpinning datasets, as is the case, for example, for QSAR models, which are documented using the QSAR model report format (QMRF), a harmonized template for summarizing and reporting key information on QSAR models including the results of any validation studies, structured according to the OECD QSAR validation principles. QMRFs are an essential part of the validation and acceptance of QSARs for use in regulatory decision making, and thus, similar approaches for DL models would be an essential next step. While the OECD have not yet developed specific guidance for DL models, they have published a set of values-based principles for the development of AI methods [147] and position papers on using AI to help combat COVID-19 [148], and on identifying and measuring developments in AI [149].

Sharing of tools and approaches, via an open innovation model, is another essential approach to achieving the promise of DL models for de novo drug design, as sharing the training set and workflow can help with understanding the computational workflow and gaining users’ trust. While this may not be entirely possible, due to privacy and policy constraints, a workaround could be the release of a “training subset” that would allow users to comprehend the model in question [150]. This can be achieved by constructing a boundary tree, based on selected training data, which is able to closely approximate the trained model. Traversing the datapoints in the tree can provide users with significantly better understanding of the model and increased trust during model sharing [150]. While some of the challenges for DL applications in medicine are related to patient data confidentiality and the need for certainty where patient care options and treatment regimens are being decided, many of the open innovation solutions currently being developed are likely applicable. First efforts towards the establishment of a DL model sharing architecture and marketplace have been demonstrated, to support the sharing of pretrained models across different ML libraries and run-time environments, with a focus on model reusability, rather than model development. In the future, standards or guidelines for model input/output format definition, as well as data mapping rules and model validation procedures, will be implemented [151].

## 11. Concluding Remarks

Since 1960, many forms of computer-aided drug design have generated a positive impact in drug discovery. Among them, structure-based and ligand-based conventional de novo drug design using evolutionary algorithms was employed for the development of novel chemical entities. AI approaches including DRL have been successfully used in the development of novel de novo drug-design approaches. Such methods include DRL using artificial neural networks including recurrent neural networks, convolutional neural networks, generative adversarial networks, and autoencoders. These methods are also used in other computer-aided drug-design approaches and, based on the promising acquired results, are expected to revolutionize the drug discovery and development process, as well as to address some of the main challenges during the early stages of drug discovery including cost and time demands, by developing in silico approaches for de novo drug design, synthesis prediction, and bioactivity prediction. Indeed, as demonstrated herein, the utility of DL-based de novo drug design for supporting drug repurposing for COVID-19 treatment has been impressive and will likely accelerate adoption of the approaches more broadly across the medical domain. The discovery of a new drug is a complex, expensive, and time-consuming process. The traditional drug development pipeline needs 12 years and 2.7 billion USD on average. The use of CADD algorithms and tools could reduce drug development costs and time significantly with conservative estimates suggesting AI pipelines require less than 1/3 of the current time and cost [152,153]. Examples of DRL-based de novo drug design include the development of adenosine A2A receptor ligands [83], rapid identification of potent DDR1 kinase inhibitors [154], and the development of a large number of new BACE1 inhibitors, which is an enzyme involved in Alzheimer’s disease [118].

Standard de novo design methods rely on the interactions with the active site of a biological target or the pharmacophores of a known active binder, and they are limited by our partial understanding of receptor–ligand interactions. DRL-based de novo drug-design approaches were developed with the goal of overcoming the limitations of existing conventional approaches. These approaches are data-driven, flexible, versatile, and can utilize a large amount of data from the scientific literature and databases. Besides the design of novel chemical entities, synthetic accessibility is also important in de novo drug design. Conventional methods partially consider the synthetic feasibility of the generated molecules based on a set of synthetic rules that are limited in a small number of retrosynthetic organic reactions [22]. DL methods allowed the development of template-free self-corrected retrosynthetic predictors to predict retrosynthesis using transformer neural networks [155].

Although the development of DL approaches in drug discovery has just begun, there is no doubt that the benefits are tremendous. However, there is still much to be done, since recent property optimization studies are focused on easily optimizable properties, such as drug-likeness [156], and efforts to integrate detailed understanding of modes of action and toxicogenomics are only beginning. Key challenges remain in terms of building community and regulatory acceptance of deep learning models, with documentation and sharing of training datasets, development of standards for model validation and model-sharing platforms as essential steps towards achieving this.

## Abbreviations

CADD	Computer-aided drug design
QSAR	Quantitative structure–activity relationships
NMR	Nuclear magnetic resonance
DNDD	De novo drug design
MCSS	Multiple copy simultaneous search
ChEMBL	Chemical database of bioactive molecules with drug-like properties
ADMET	Absorption, distribution, metabolism, excretion, and toxicity
AI	Artificial intelligence
ML	Machine learning
DL	Deep learning
RNN	Recurrent neural networks
CNN	Convolutional neural networks
GAN	Generative adversarial networks
AE	Autoencoders
RL	Reinforcement learning
DRL	Deep reinforcement learning
SMILES	Simplified molecular-input line-entry system
ReLeaSE	Reinforcement learning for structural evolution
TL	Transfer learning
LSTM	Long short-term memory
nll	
2D	Two-dimensional
DNN	Deep neural network
RANC	Reinforced adversarial neural computer
ATNC	Adversarial threshold neural computer
IDC	Internal diversity clustering
VAE	Variational autoencoder
3D	Three-dimensional
MW	Molecular weight
LogP	Octanol-water partition coefficient
HBD	Hydrogen-bond donor
HBA	Hydrogen-bond acceptor
TPSA	Topological polar surface area
seq2seq AE	Sequence to sequence autoencoder
GRU	Gated recurrent unit
AAE	Adversarial autoencoder
PSO	Particle swarm optimization
OECD	Organization’s for the Economic Cooperation and Development
SA	Synthetic accessibility
SC	Synthetic complexity
MOA	Mechanism-of-action
COVID-19	Coronavirus disease 2019
SARS-CoV-2	Severe acute respiratory syndrome coronavirus 2
M^pro^	Main protease
ACE-2	Angiotensin II
WGAN	Wasserstein GAN
US FDA	United States food and drug administration
GCGR	Glucagon receptor
EMA	European medicines agency
HMA	Heads of medical agencies
QMRF	QSAR model report format
DDR1	Discoidin domain receptor 1
